# Lived experiences of recovery following musculoskeletal trauma 6 months following injury in the UK: a qualitative study

**DOI:** 10.1136/bmjopen-2025-108425

**Published:** 2025-11-19

**Authors:** Nicola Middlebrook, Nicola R Heneghan, Maria Moffatt, Lucy Silvester, Deborah Falla, Alison B Rushton, Andrew Soundy

**Affiliations:** 1 Department of Health Professions, Manchester Metropolitan University, Manchester, UK; 2 School of Sport, Exercise and Rehabilitation Sciences, University of Birmingham, Birmingham, UK; 3 School of Medicine and Dentistry, University of Lancashire, Preston, UK; 4 Institute for Applied & Translational Technologies in Surgery, University Hospitals Coventry and Warwickshire NHS Trust, Coventry, UK; 5 Centre of Precision Rehabilitation for Spinal Pain, School of Sport, Exercise and Rehabilitation Sciences, University of Birmingham, Birmingham, UK; 6 School of Physical Therapy, Western University, Faculty of Health Sciences, London, Ontario, Canada

**Keywords:** Musculoskeletal disorders, QUALITATIVE RESEARCH, REHABILITATION MEDICINE

## Abstract

**Abstract:**

**Objectives:**

Traumatic musculoskeletal injuries are common and often life changing. The 6-month period following injury is a critical phase in recovery, during which engagement with rehabilitation professionals can be key to achieving positive outcomes. However, there is limited understanding of which aspects of recovery matter most to patients, how they define a successful recovery and what this looks like when captured during the lived recovery process. The aim of this study was to explore patients’ views and perceptions of recovery following musculoskeletal trauma and to understand what constitutes successful recovery at 6 months postinjury.

**Design:**

Qualitative study using interpretative phenomenological analysis through semi-structured interviews.

**Setting:**

Major trauma centre in the UK.

**Participants:**

13 participants (mean age (SD) 51 (17) years, 69% male) completed the interview. Eligibility criteria: adults >18 years, musculoskeletal injury from a traumatic event and ability to communicate in English. Purposive sampling included age, gender, injury severity and injury type. Interviews were audio recorded and transcribed verbatim.

**Results:**

Three main themes were identified: (1) The recovery journey: participants reflected on their recovery while still processing the accident/injuries. They often drew on other people’s experiences to make sense of their recovery. Recovery was accompanied by a range of negative emotions and personal challenges. (2) The outcome: participants used multiple strategies to work towards recovery, guided by personalised individual goals. A successful recovery was defined as their ‘normal’. (3) Healthcare/clinicians impact recovery: Participants reported confusion and mixed messages from healthcare professionals, leading to uncertainty around injury management. Fragmented care pathway and difficulties accessing healthcare and support were also highlighted.

**Conclusions:**

Recovery at 6 months post injury involves a complex interplay of physical and emotional factors. This period can be particularly challenging to navigate, often with no or limited support. There is a need for a targeted, individualised approach to guide patients through this period of recovery. Participants’ focus was on the outcome following their injury, setting goals for the future, with successful recovery defined as ‘normal’. Further research is needed to support clinicians in developing effective psychologically informed rehabilitation plans.

**Strengths and limitations of this study**

- Interpretative phenomenological analysis was employed, enabling a rich and in-depth exploration of the participants’ recovery experiences and the meanings attributed to their recovery journeys.
- This study is reported in accordance with the Consolidated Criteria for Reporting Qualitative Research, ensuring transparency and rigour.
- Multiple strategies were used to enhance trustworthiness, including blind reviewing of data, member checking and a collaborative approach to data analysis.
- The researchers who interviewed and completed the initial stages of data analysis had similar experiences and backgrounds which may have influenced the interpretation of the results.
- Data were collected from a single region within the UK, which may limit the transferability of findings to other contexts.

## Introduction

 A total of 500 000 people in England experience traumatic injuries annually, with over 45 000 sustaining severe injuries.^1^ Types of injuries include fractures, soft tissue injuries and limb loss.^1^ Advances in healthcare have increased survival rates, leading to a greater need for rehabilitation.^1 2^ Specialist rehabilitation often focuses on severe and complex injuries, but less severe injuries also frequently require a period of rehabilitation. Irrespective of injury severity, recovery can often extend up to 2 years postinjury.^1 3 4^

Following injury, individuals often experience significant long-lasting changes that impact their quality of life and psychological well-being.^4^,^10^ Multiple studies have explored patient experiences of rehabilitation and recovery at various stages, focusing on subgroups of patients such as those with ankle fractures,^11^,^14^ major traumatic injury^15^,^18^ and humeral fractures.^19 20^ Key findings highlight service-wide issues in accessing healthcare for rehabilitation, miscommunication or mixed messages from healthcare staff.^15^,^1721 22^ Individual findings of these studies highlight both physical and psychological effects and barriers to accessing healthcare. Physical impacts include loss of independence, restricted activities of daily living (ADL), pain, poor sleep and financial/work implications.^11^,^13^ Psychological impacts include fear of reinjury, burden on others and adjusting to life with an injury/impairment.^12^,^1419^ Evidence suggests that prolonged distress, anxiety and catastrophising can predict poorer outcome long term.^423^,^25^ While changes to mood and emotion are expected following an injury,^1 26^ these features can become predominant in later stages of recovery, leading to reduced participation in activities, low mood and social isolation.^1^ It is, therefore, important to explore patient views at later stages of recovery (eg, 6 months) to inform and implement strategies that improve long-term outcomes. Studies often include participants at different stages of their recovery, such as within the first year of injury. While this provides an overview and range of perspectives, it limits the opportunity to gain a deeper understanding of the lived experience at that moment in time and, therefore, does not fully represent their experience.

6 months following injury is a critical point in the recovery process, as most individuals have been discharged from hospital and are actively engaging in rehabilitation. Evidence suggests that this is the stage when many individuals begin to see improvements in function and pain,^27 28^ making it a suitable timepoint for capturing experiences than earlier stages in recovery such as 3 months, which align more closely with healing timelines. Furthermore, research suggests that injury severity and healing times do not necessarily correlate with rehabilitation needs or recovery trajectories.^1 29^ Recovery can extend beyond 24 months, particularly for those with complex rehabilitation needs, with many not returning to preinjury levels of function or returning to work.^6 10 27^ Poorer function and higher pain intensity at 6 months have been suggested to predict poorer outcomes long term,^4^ as well as the potential for psychological features such as anxiety or low mood becoming predominant in later stages of recovery.^1^ This highlights the importance of optimising rehabilitation 6 months postinjury. Rehabilitation should be tailored to the individual, taking into account their needs, pre-existing conditions and contextual factors such as psychological distress or social and financial circumstances.^1^ Nonetheless, there remains a lack of clarity regarding which aspects of recovery patients deem important, how they conceptualise a successful recovery and how their experiences—typically elicited through retrospective accounts rather than captured during the lived recovery process—can be effectively understood at the 6 month postinjury stage.

There is, therefore, a need to explore the lived experience of patients with varying injury severities at 6 months postinjury to understand their views on recovery and what successful recovery means to them. This information may help tailor rehabilitation to meet patient needs and improve long-term outcomes. It can also guide trauma services in targeting efforts to support patients in their recovery. The aim of this qualitative study was to explore patients’ views and perceptions of recovery and what constitutes recovery at 6 months following traumatic musculoskeletal injury.

## Methods

### Design

This study forms part of a larger study where patient views and experiences were followed longitudinally over three interviews (within 4 weeks of injury, at 6 months and 12 months) with a predefined and published protocol.^30^ Findings focusing on views and experiences within 4 weeks of injury have been published.^29^ The methodology used for this study was interpretative phenomenological analysis (IPA) using semi-structured interviews. IPA assumes a world view of minimal hermeneutic realism. This view identifies that external reality exists, but the exact meaning is provided or constructed by an individual.^31^ This study is reported according to Consolidated Criteria for Reporting Qualitative Studies (online supplemental file 1).^32^

### Reflexivity

Authors NM and MM who undertook the interviews were physiotherapists and had experience of post-doctoral qualitative research. Both authors were familiar with the topic and undertook interviews at 4 weeks following injury.^29^ Participants did not know the authors prior to participating in the study but understood the nature and aims of the research.

### Participants

Inclusion criteria comprised adults (>18 years), who had sustained a musculoskeletal injury from a traumatic event and had mental capacity (score of more than 6 on the Abbreviated Mental Test),^33^ and could communicate in English. Adults who had experienced an injury where the primary injury was a traumatic brain injury, spinal cord injury or neurological injury were excluded.

### Sampling and sample size

All participants (n=17) who completed the first interview at 4 weeks following injury were invited to complete the 6-month interview. Purposive sampling for the 4-week interview was employed, including age, gender, injury type, severity and location of injury.^29^ Sample size considerations were based on principles of IPA which required typically low numbers; this is often between 1 and 30, but more often limited to between 6 and 20 in past text.^34^

### Data collection methods

Semi-structured interviews were conducted with a topic guide developed by the research team and patient coinvestigator using knowledge from a previous study^4^ and informed by the International Classification of Function, Disability and Health domains.^35^ A copy of the topic guide is provided in online supplemental document 2. Questions were focused on their experience of recovery, long-term and short-term goals, exploring factors which potentially hindered or helped them, their definition of successful recovery and interactions with physiotherapists in their journey so far. All interviews were audio recorded and transcribed verbatim. Field notes and a reflexive diary were completed to enhance trustworthiness of the data. All participants were given the opportunity to review the transcript and provide additional comments/further insight following the interview.

### Setting

Participants were recruited from one Major Trauma NHS Trust site within the UK. This hospital covers a wide area which includes both urban and more remote rural areas. The semistructured interviews were conducted online via Microsoft Teams or a telephone call.

### Data analysis

Analysis was undertaken using a four-stage approach designed for IPA studies.^36^

Stage 1: Transcripts were read independently by NM and MM.

Stage 2: Preliminary themes were identified by NM and MM independently and coded. This was to independently identify understanding of the individual’s own interpretation of their experience but also identify the author’s interpretation, achieving the double hermeneutic required for IPA studies. Preliminary themes were then discussed with AS, who acted as a critical friend during initial coding.

Stage 3: NM grouped emerging themes and were presented in a summary table with verbatim abstracts. Themes were critically discussed with co-investigators (ABR, AS, DF and NRH).

Stage 4: A summary table was presented (and critically discussed) to the study steering group (NM, patient and public involvement (PPI) coinvestigator, LS, AS and independent chair). This occurred on three occasions in an iterative process where themes were challenged, changed and modified in response to the steering group. Specific words, extracts and meaning were presented to the group across the whole themes to achieve the hermeneutic circle as required in IPA studies.

#### Injury severity

The Injury Severity scale is a retrospective tool used in a clinical setting where injury severity is rated using a numerical scale and was used to categorise injury severity of participants. 0–8 is characterised as mild injury severity, 9–15 moderate and 10–24 major severity. A score over 25 indicates a profound injury.^37 38^

### Transparency and trustworthiness of findings

Multiple strategies were employed to ensure trustworthiness.

1. Blind reviewing of the data in Stages 1 and 2 by NM and MM.
2. Presentation of the themes throughout analysis to both the Study Management Group and the Study Steering Group allowed peer and patient critique and a collaborative approach to data analysis.
3. Acknowledging lead researchers’ potential preconceptions and beliefs and encouraging reflexivity enabled transparency.^39^

### Patient and public involvement

Patient and public involvement (PPI) has been integral to this project with a PPI coinvestigator from inception. PPI involvement was included within the development of the study, topic guide development and the final analysis stages to improve interpretation and presentation of themes. Details around how the study has been informed and developed can be found in the published protocol.^30^

## Results

### Participants

13 participants completed the 6-month interview. Mean (SD) age of participants was 51.46 (17.48) with 9 males and 4 females. Mechanism of injuries included road traffic collisions (n=6), fall of more than 2 m (n=3), fall of less than 2 m (n=1), sporting injury (n=2) and crush injury (n=1). Participant characteristics are summarised in table 1. One participant completed the interview on the telephone (Microsoft Teams was inaccessible), and the reminder of interviews was completed on Microsoft Teams. Only the researcher and participant were present for the interviews. Interviews lasted between 30 min to 1 hour.

**Table 1:** Participant characteristics

Participant	Mechanism of injury	Injury severity score and category
P001	Road traffic collision	Moderate
P004	Fall more than 2 m	Moderate
P007	Crush injury	Major
P008	Fall more than 2 m	Moderate
P010	Sporting injury	Moderate
P012	Road traffic collision	Mild
P014	Fall less than 2 m	Mild
P015	Road traffic accident	Moderate
P017	Fall more than 2 m	Moderate
P018	Sporting injury	Moderate
P020	Road traffic accident	Mild
P021	Road traffic accident	Moderate
P024	Road traffic accident	Major

### Emerging themes

**Figure 1:**
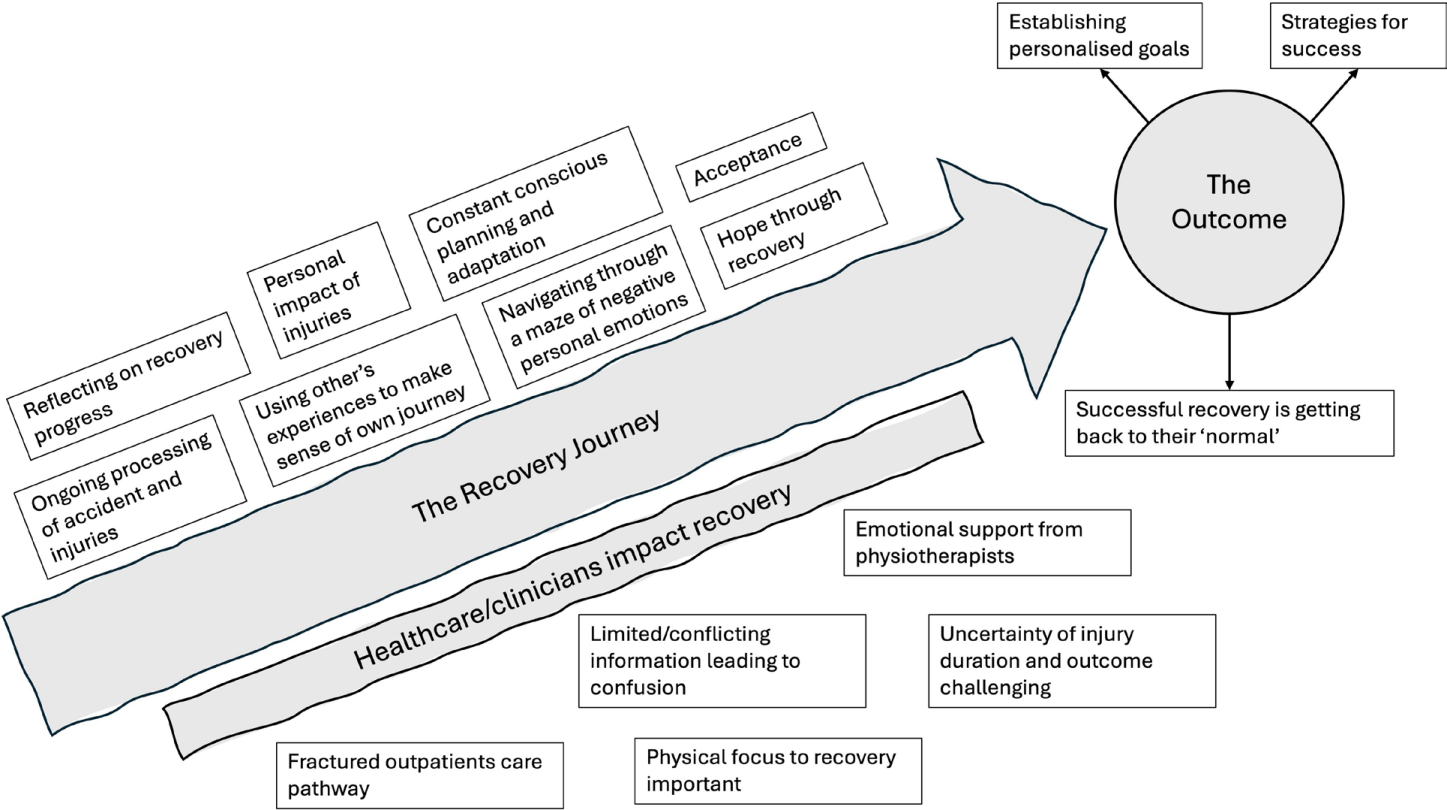
An illustration to demonstrate the journey of recovery at 6 months. Grey illustrates the main themes with subthemes in white.

Three main themes emerged: (1) the recovery journey, (2) the outcome and (3) healthcare/clinicians impact recovery. All participants were continuing their recovery journey irrespective of type of injury, mechanism of injury or injury severity. There was a wide range of emotions and processing of the accident/injuries/rehabilitation and recovery. Multiple influences were identified on the recovery journey. Figures1  2 summarise the key themes/subthemes.

**Figure 2:**
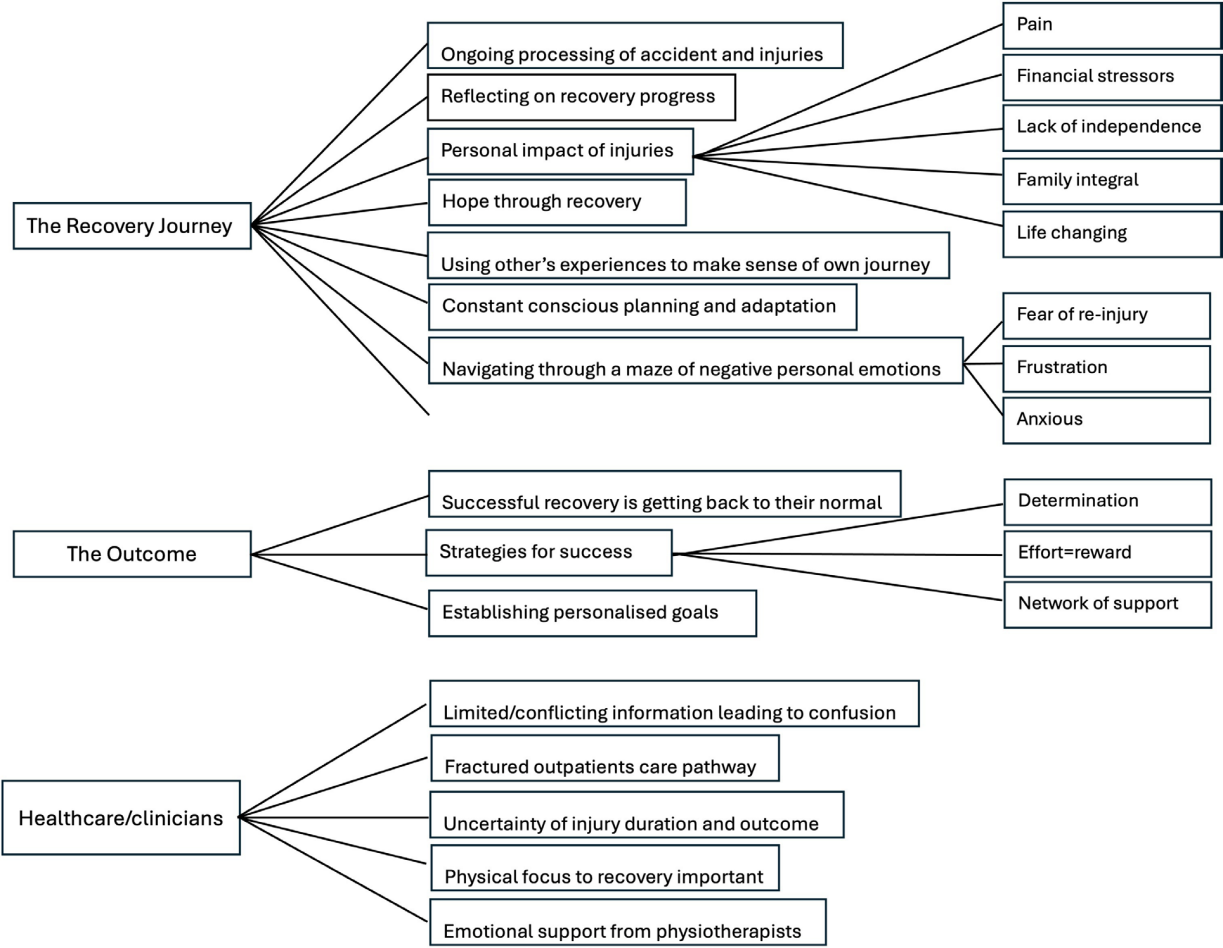
Coding tree of themes and subthemes.

#### The recovery journey

Participants reflected on their recovery progress and spent a considerable amount of time discussing and processing the accident and injuries and what had happened to them. They used other people’s experiences to try and make sense of their own injuries and recovery. Their injuries had a negative impact including pain, financial stressors, lack of independence and impact on their family. This was intertwined with trying to navigate through a maze of negative emotions such as frustration, fear of reinjury and anxiety. Participants described a feeling of constant planning and adaptation because of their injuries but had started to accept the injuries and current function level and felt hope through their recovery journey.

##### Reflecting on recovery progress

Participants reflected on their progress with each participant at a different stage in their recovery. Some participants were focused on specific functional tasks:

I can actually put some weight down on the foot and rest the foot down. And they’ve given me an air cast boot. So, I can start putting weight on it and stuff. So, I’m now on crutches. No, I wouldn’t say walking, but I’m just obviously being able to manoeuvre a little bit better. So, it’s been a little bit better for spirits P015 [Male, 30-39]

While others reflected on their journey more generally:

But now, I would say I’m probably 95% recovered. So, really doing, like, I’m back horseback riding; I’m back, you know, pretty much doing the same level of physical activity as before: hiking, whatever. I think that side is still a bit weak… weaker than the other side. But I’d say, like, again, 95%, you know, pretty much back to normal P004 [Female, 30-39]

##### Ongoing processing of accidents and injuries

At 6 months after their injuries, participants were still processing the injuries and accident itself, trying to understand why it had happened:

I went to speak to the three of us on the trip. The one chap was behind me, obviously he saw exactly what happened, saw the car coming round the bend, come onto my side of the road and hit me. And that is the point where I’ve lost all memory, if you like P012 [Male, 70-79]

Other participants described the process of how they were understanding their injuries and how specialist support had helped in processing their accident and recovery:

The hospital arranged a counsellor, and she told me a lot about the three parts of the brain……. effectively, you’re grieving—no different to a death in that something that you had—and obviously getting your head around how it might have turned out P008 [Male, 50-59]

##### Using others’ experiences to make sense of own journey

Participants detailed how they talked to others with similar injuries to understand their own recovery journey and their prospects in returning to normal:

Speaking to other people, especially in the motorcycle industry, sport, whatever you want to call it, I’ve spoken to people that have had similar injuries and one of them, he had his accident 20 years ago and he’s pretty well back to normal. I spoke to another guy who had a similar accident from 2014, eight years ago, and he’s completely back to normal P001 [Male, 30-39]

So, you know, the next six months, really, are sort of going to tell me whether I’m going to make a complete recovery or there might be just sort of some minor ailments in the cold weather or something like that. I mean, I’ve spoken to a colleague of mine at work, another lorry driver and he’s had similar surgery and he’s saying the cold weather affects him and stuff like that. If he does a lot of walking or does too much, he notices it. So, we’ll see. So, that’s where I’m at P020 [Male, 50-59]

##### Hope through recovery

Participants expressed hope for recovery using their progress as a guide. Some participants were specific in describing this:

I’m hoping, yeah, just be my strength and everything will be there. I won’t have to worry about bashing it, it won’t be in my mind, it’ll just be the metal in my leg P018 [Female, 18-29]

Other participants were more generic in describing this:

I think that I will have made 80% to 90% recovery, I think and I’ll be walking a lot better and just be back to my normal job. And there might just be some minor ailments with the injury long-term. That’s what I foresee P020 [Male, 50-59]

##### Personal impact of injuries

The injuries impacted individuals in various ways, including pain, financial stress, loss of independence, impact on their families and that the experience had been life changing. The impact was individual to the participant and their life at that moment in time. Pain was described as impacting the activities they could do:

The pain and the silly things that… how so little it takes to make it really painful…… Yeah, obviously, I’m right-handed, so there’s so many things that I’ll just go to do, and then I have to stop and change tack really P008 [Male, 50-59]

Financial stressors and navigating the complex income support system were described particularly as taking up time:

I am still having sort of like little “dances” with the mortgage people. Well, they’ve been pretty good, I suppose. I saw a budget planner as well and I said, “Look, this is like… once it all gets sorted, hopefully I will be on the phone straightaway and I will be paying you off, you know. That could be like a year, two years, maybe something like that P007 [Male, 50-59]

Lack of independence was described as the impact this has on daily activities:

Sometimes going to places without a bike can be very difficult, especially in the middle of nowhere. Where the bus literally only comes twice a day. And then you have to walk quite a long time to get to that, to where you want to and that is just not possible P014 [Female, 70-79]

Family was described as integral to their recovery but that it has been challenging for them:

It’s been a difficult time. My brother and partner have been fantastic, but I know it’s been a strain on them P017 [Male, 50-59]

And participants described the whole experience as having been life changing:

It’s definitely life changing and it’s definitely… it’s a real weird thing. Part of me is not happy, but it’s changed a lot of things and put things into perspective. It’s been good and it’s been bad. I like what has happened from that, but it’s been tough. It has been tough. When I spoke to you guys last, I was in a good space. Coming over Christmas I wasn't in the best; February I wasn't the best P001 [Male, 30-39]

##### Constant conscious planning and adaptation

Participants reported the feeling of having to plan everything consciously which was often described as being exhausting in itself:

I think it’s just planning really, just making sure that things are planned properly. I mean you get tired just thinking about these things, just to make sure, is that right, is that right P010 [Male, 40-49]

Some participants discussed the conscious planning as a risk assessment before executing an activity. These activities were day-to-day activities which were previously automatic, and participants described this conscious planning with the majority of their daily activities:

It’s just been thinking of how we’re going to do this. How we’re going to be able to do that? Who’s going to be able to be there to help me? Or is it worth me risking going downstairs to take the trash out and then walking across like on the side of the road? P015 [Male, 30-39]

Others described how adapting is now taking much longer to do activities:

Before just to get to the supermarket, I just swung myself on my bike and I was there in about three minutes. And now at first, believe it or not, just to go down the road, it took me two hours to go about 300m or so P14 [Female, 70-79]

##### Navigating through a maze of negative personal emotions

Participants described various negative emotions such as frustration, fear of reinjury/setbacks and anxiety. These intertwined emotions are often linked to other subthemes including personal impact of the injuries, acceptance, uncertainty around recovery duration and processing of the accident and injuries. Emotions were unique to each individual and their own recovery journey:

I mean at the moment very frustrating because I’d literally just gone back to it, ready to crack on and it is frustrating, but I can’t do much about it now other than try and get it back to how it was P018 [Female, 18-29]

I’m conscious of pushing myself too far and doing more damage. That is what I am fearful of, because I don’t know how far to push it because I’ve never had this before so I can only go with what I’m comfortable with, I guess P010 [Male, 40-49]

I’m more anxious than I was in that I don’t want any stress; I’m not prepared to put up with any stress P008 [Male, 50-59]

##### Acceptance

Some participants acknowledged that they might not fully regain their previous level of function. Some had progressed further in accepting this reality than others:

I accept that I’m not going to be able to walk the same as I used to. My arm is metal pretty well all the way through, screws and metal joints. So that’s never going to be quite the same as it was. I accept that. But I can get them to a pretty good standard. If I can just work on them P012 [Male, 70-79]

The knee is probably the most painful and there may be nothing we can do about that, it’s just functionally not a normal knee joint but it is astonishing it is there at all and I can walk well on it P024 [Female, 40-49]

### The outcome

Participants described using multiple different strategies to help them in their recovery, including determination, the notion of more effort means more reward (better recovery) and using their network of support to help them recover. Successful recovery was described as ‘normal’, but normal was individual to every participant and was often referred to how they were pre-accident, irrespective of injury type or severity. Individualised goals had been established, and this was individual to their current function level and working towards their overall goal of getting back to their normal.

#### Strategies for success

Participants employed various strategies for success which were specific to each participant and their individual circumstances. Strategies included intrinsic factors such as determination, the notion of more ‘effort=reward’ and extrinsic factors such as utilising their network of support which could include healthcare, family and friends:

Probably more my mindset, if I can refocus it… it won’t feel as challenging as it is right now, and how it has been the last two weeks P018 [Female, 18-29]

I’d say if I get my finger out and do the physio, that will be the key to getting back as much as I can P017 [Male, 50-59]

Of course, I must mention my wife, you know, because she has been a great help to me through all this. She’s really helped me out a lot. So, I’m very grateful for that P020 [Male, 50-59]

#### Successful recovery is getting back to their normal

Despite being able to accept that full function might not be achievable, successful recovery was described as returning to ‘normal’ referencing their pre-accident state. More emphasis was placed on activities rather than specific issues such as pain or walking. ‘Normal’ was unique to everyone and related to their life rather than a generic definition. Injury severity did not appear to alter the definition:

I don’t like hobbling about……I like to be doing, I like to be out in the garden, if I’m not gardening, I’m doing something, I like generally to be busy and doing……I just want to move forward with all that sort of thing and then that will give me more of a normal life P012 [Male, 70-79]

I would say getting back to whatever you were doing before, the same. No incremental physical limitations. That’s what I would say “recovery is” P004 [Female, 30-39]

To be as I was before the accident really……Well, for right now, I just think “recovery” means as good as was before…. “recovery” for me means to get back as to how you were before and stuff. Anything less is not going to be recovery for me P007 [Male, 50-59]

#### Establishing personalised goals

Every participant had goals in their recovery journey which were related to their definition of successful recovery. These goals were personalised to each individual’s circumstances and current stage of recovery:

But getting back to that (work) would be good. Just to get back to normality. As I say, it’s all right being at home every day, but it isn’t, if you know what I mean? P007 [Male,50-59]

Yeah, I just want to be able to be fit again. Just regular stuff. Just being able to get up out of bed and not feel sore. Just normal stuff. Getting out of the chair. Walking up stairs. Going to the toilet. Going to have a shower. Going to the fridge. Just normal stuff P001 [Male, 30-39]

### Healthcare/clinicians impact recovery

Participants often described limited or conflicting information given to them by clinicians and this led to confusion. There was uncertainty about their injuries and the long-term outcome, and this was challenging to process. There was difficulty in accessing care and, when accessed, the outpatient care pathway was not fit for purpose. When able to access support from physiotherapy, it was highlighted that emotional support was valued, but the focus was physical recovery, which was important to them.

#### Limited/conflicting information leading to confusion

Participants faced difficulties accessing information during recovery and often received conflicting advice from healthcare clinicians. This limited and conflicting information led to confusion, complicating their efforts to understand their injuries and being able to navigate their recovery journey effectively:

At the time, when I did initially get discharged, I thought I was going to be okay and that was it. Then it was, “No, you’re not. Your bones are quite bad so we’re not going to be able to fix that.” Then it went, “No, we’re not going to be able to fix that.” So it was a bit of a rollercoaster of 'we’re going to fix this, no we’re not, yes we are, we can do this, no we’re not.’ That is probably the bigger things I’ve had P001 [Male, 30-39]

I don’t know what to expect. No one’s really said to me what I can expect. I expect I might have a limp; I don’t know. Again, no one’s had that kind of detailed conversation with me really P010 [Male, 40-49]

#### Fractured outpatients care pathway

Participants highlighted the challenges in accessing care and support, particularly in the first 3 months after discharge when they felt it was most needed. Some reported little to no support, describing it as ‘falling off a cliff’ after discharge with participation in a research study being their only source of support:

I think some of the aftercare, if you like, could be a little bit better, I think. There isn’t really anything for… apart from talking to you guys (researcher), there isn’t really any kind of people there for you. It’s up to you really to sort it. And I’m sure that some people, if they haven’t got a family or something, it must be hard…… Yeah, falls of a cliff a bit, yeah. So I think if you’ve got support at home, like I have, then that makes a big difference P012 [Male, 70-79]

If they could access care, it was often ‘fractured’ and not fit for purpose due to the demands on the Health Service:

A lot of physio appointments. Well they’re not really physio really, not what you’d term. Everyone says, oh you’re going to physio two or three times a week. It doesn’t really work like that. It’s every eight weeks. She assesses me on my strength really and how well I am standing and then she will give me more or carry on with the same exercises P010 [Male, 40-49]

#### Uncertainty of injury duration and outcome is challenging

Participants expressed uncertainty about their recovery duration and their overall outcome and how long it will take to achieve a full recovery. They describe recovery in months and years using guidance from healthcare professionals which was frequently unclear and lacked definitive answers:

I am probably concerned about the last time I went to the hospital, it was a consultant who has been like a support consultant, standing in, so he had no idea of history etc. and he said, oh the swelling is taking quite a long time. The bones kind of really sort of worry me really. I said, is it anything to be really concerned about? He says, no, I don’t think so, it’s just the fact that you’ve broken quite a few bits in there P010 [Male, 40-49]

Well, my hope and belief was about six months I’d be up, back to normal, bouncing around, you know, and that isn’t happening. But, I mean, when I first spoke to the surgeon when I had the surgery, I said, “How long? I’m a lorry driver and I want to go back,” I said, “how long?” She said, “Oh, 12 months.” She said, “Or, maybe, six months. P020 [Male, 50-59]

#### Physical focus to recovery important

Although emotions were described in previous themes, participants spoke about the importance of the physical aspect of recovery and rehabilitation being integral to this:

Yeah, especially with the whole muscle manipulation, it did really make a massive difference. Because my back was so messed up from the crash. Obviously if I didn’t see an osteopath before, I would have just been trying to walk properly with my back all trying to pull me to one side. So yeah, definitely is a major part P018 [Female, 18-29]

#### Emotional support from physiotherapists

Although the physical aspect of recovery was important, participants acknowledged that physiotherapists gave valuable support emotionally in their recovery:

The one (physiotherapist) I have now is an amazing therapist as well, she’s incredible at helping with my mental and general, just general health. They sort of set you small increments of improvement which is good, it gives you the chance to achieve that without feeling frustrated that you haven’t achieved anything bigger. I found every physio I’ve met very helpful and kind and supportive P024 [Female, 40-49]

## Discussion

The aim of this study was to explore patients’ experiences 6 months after musculoskeletal trauma. Key findings reveal that recovery involves a complex interplay of physical and emotional factors. While healthcare support is crucial and actively sought, patients often experienced it as limited, disjointed and inaccessible (eg, no appointments or long waiting times). Conflicting information from healthcare providers hinders patients’ ability to progress and understand their recovery, often leaving them confused. Regardless of injury severity, patients desire to return to their preinjury ‘normal’.

### The recovery journey

This theme highlights significant emotional burden/distress experienced by participants 6 months after injury. Previous studies support these findings, particularly around the impact of injury^11^,^1319 21^ and psychological effects.^11 13 15 19 40 41^ However, this study identifies some new findings such as continued processing of injuries and the accident. Other themes such as conscious adaptation and planning and acceptance have been previously reported within the literature^18^; however, this study has given new insight into how these factors are still present in later stages of recovery and how this impacts on their recovery journey and interacting with others such as therapists and family. This, in combination with previous findings, gives a greater understanding of the complex interplay of multiple factors which are contributing to the recovery journey and highlights that, at 6 months following injury, the emotional distress is significant. This adds to the understanding of the potential causes of emotional burden, the distress that participants experience and crucially how these insights could help target interventions and screening to enable patients to manage or regulate their emotions.

While distress following an accident is expected in the initial weeks following an injury,^1 26^ the findings suggest that distress/emotional burdens continue beyond the initial stages of injury. Higher levels of distress following injury can result in poorer outcomes and development and maintenance of long-term pain.^25 26 42^ Most literature, however, is focused on mild musculoskeletal injuries such as whiplash, indicating a need for further research on more complex musculoskeletal injuries. Emotional distress can potentially affect rehabilitation engagement, making identification of distress and subsequent support to the patient critical.^43^ Psychologically informed care postdischarge could help patients manage their emotion/distress by developing strategies as their recovery progresses and thus improving rehabilitation engagement and long-term outcomes.^43^,^46^ Further research is needed on the growing body of literature of a stepped early psychological and psychosocial care pathway which starts within the hospital setting but continues into the community setting.^47 48^

### The outcome

This theme reveals participants are focused on returning to their ‘normal’ and use various strategies to achieve this. Previous studies suggest that rehabilitation priorities and definitions of successful recovery evolve over time to a ‘new normal’.^18^ However, this study finds that 6 months postinjury, participants often continue to define ‘normal’ in reference to their preinjury state. This stands in contrast to the subtheme of ‘acceptance’ also identified in this study which reflects a recognition that full functional recovery may not be attainable. Participants may be in the midst of a transitional process involving understanding, adaptation and adjustment^18^ during which their perceptions of recovery and what constitutes ‘normal’ are still evolving. It is imperative that clinicians support patients in navigating this period with an individualised approach dependent on their stage in this process.^18 48^ Effective communication around guiding patients understanding, their management of emotions and distress and patient empowerment is crucial. Use of frameworks such as developing therapeutic relationships used in chronic musculoskeletal pain may be useful here to support the therapist.^49^ However, adequate training as well as changes within the physiotherapy service or department needs to be considered for this approach to be effective.^44 50^

### Healthcare/clinicians impact recovery

This theme demonstrates the important role clinicians play in the recovery journey both in the acute phase but also in later stages of recovery. Participants consistently sought guidance from healthcare providers to understand their recovery but faced issues with accessing healthcare, conflicting or limited information. Within the UK, healthcare is accessed mainly through the National Health Services compared with private healthcare, making the results of this study unique to the UK. However, previous research from both the UK and other countries has highlighted poor communication as a barrier to recovery.^15^,^1722^ Effective communication is vital for developing therapeutic relationships providing psychologically informed care.^45^ This study highlights shortcomings in accessing care and poor communication leading to uncertainty around recovery. Improving patient care through the trauma recovery pathway, from discharge to community-based rehabilitation, is essential. This should include enhanced training for healthcare professionals in effectively communicating how to manage uncertainty in their recovery journey.

Although psychological distress and negative emotions have formed a large part of the findings, participants also valued a focus on their physical injuries. Rehabilitation should prioritise physical injuries and restoration of function together with psychological interventions, which are individual and meaningful to the patient.^51^ This highlights the need for further research to develop outcome measures or a core set of measures for this population.^18^

### Strengths and limitations

The strengths of this study are that it explored a breadth of injury severities rather than a focus on one specific injury representing clinical practice. It captured the lived experience at 6 months rather than a reflection of their recovery, offering insight into mindsets and challenges at that moment in time. Capturing the lived experience at that significant point allows insight into their perceptions, making sense of their recovery journey as it is happening and also reflecting a more accurate representation of the challenges which healthcare professionals may encounter. There are, however, some limitations. This study was conducted in one region of the UK, therefore, it cannot be representative of perspectives from participants in differing parts of the UK, which may have different access to services which could influence patient recovery. This, therefore, limits the transferability of the study to the wider population but adds greater insight into patient experiences, which is important to understand and can inform and influence clinical practice and service development. While steps were taken to ensure trustworthiness, inclusion of other health professions in the analysis and reflexivity of the research team rather than the two members of the team would have further enhanced the rigour of this research.

## Conclusions

This study has demonstrated a complex interplay of physical and emotional factors at 6 months postmusculoskeletal injury. This period of recovery is challenging for patients to navigate and support from healthcare professionals is actively sought. However, support was limited, disjointed or inaccessible. There is a focus on the patient outcome following their injury, setting goals for the future, with successful recovery defined as ‘normal’. There is a need for a targeted, individualised approach to support patients during this period of recovery. Further research now needs to focus on supporting clinicians to develop effective communication and deliver psychologically informed care to support patients in their recovery.

## Supplementary material

10.1136/bmjopen-2025-108425 online supplemental file 1

10.1136/bmjopen-2025-108425 online supplemental file 2

## Data Availability

All data relevant to the study are included in the article or uploaded as supplementary information.
