# Supplementary material for: Lived experiences of recovery following musculoskeletal trauma 6 months following injury in the UK: a qualitative study
Source: BMJ Open. 2025 Nov 19;15(11):e108425. doi: 10.1136/bmjopen-2025-108425 (PMC12636951; doi:10.1136/bmjopen-2025-108425)
Supplement: Supplementary file 3 [file bmjopen-2025-108425.reviewer_comments.pdf]

PEER REVIEW HISTORY

BMJ Open publishes all reviews undertaken for accepted manuscripts. Reviewers are asked to complete a checklist review form and are provided with free text boxes to elaborate on their assessment. These free text comments are reproduced below.

ARTICLE DETAILS

Title (Provisional)

The lived experiences of recovery following musculoskeletal trauma 6 months following injury in the United Kingdom-a qualitative study

Authors

Middlebrook, Nicola; Heneghan, Nicola R; Moffatt, Maria; Silvester, Lucy; Falla, Deborah; Rushton, Alison B; Soundy, Andrew

---

VERSION 1 - REVIEW

**Reviewer**	**1**
**Name**	**Tsagkaris, Christos**
**Affiliation**	**University of Zurich**
**Date**	**07-Sep-2025**
**COI**	**None**

The authors have explored patients' experience in the process of (major) injury recovery in a qualitative manner. Although this design does not allow collecting evidence from a larger number of patients, it is indeed useful in terms of defining key themes, that can be further searched for in a quantitative manner. The paper has therefore merit and contributes to the understanding of patients' expectations and experience in the short term after injury. The following points could be addressed to provide more clarity:

1. Why is the time interval of 6 months decisive according to the authors? One could argue that a time interval of 3 months - estimating 6 weeks of fracture healing and 6 subsequent weeks of gradual mobilisation could also be considered a turning point.

2. Limited demographic information about the participants has been provided - certainly their age and biological sex distribution as well as type of injury are documented, however it would be helpful if the authors could provide more information enabling the reader to contextualise the social history and determinants of health (or disease) among the patients - eg their working status and whether they are in permanent or temporary employment, their residence in an urban/rural environment, their family status. These are representatives examples, but it may be undertstandable that data potentially leading to the identification of the patients cannot be presented.

3. The issue/theme of financial (in)security has appeared. Discussing the insurance and social protection context in which the system supports patients would be useful for the reader - does for instance the inability to afford mortgage payments indicate that the salary of the patient was not (fully) subsidized or that the subsidy was consumed in the form of out-of-pocket payments, that could not be addressed in the frame of formal direct and indirect healthcare expenses?

**Reviewer**	**2**
**Name**	**Tang, Clarice**
**Affiliation**	**Victoria University**
**Date**	**19-Sep-2025**
**COI**	**None**

The manuscript covers a topic that is relevant and will be of interest to health professionals who worked with traumatic msk injuries. Considering the amount of current literature focusing on this topic, there appears to have quite a bit of published literature around emotion and physical factors influencing optimal rehabilitation at 6 months post injury. In addition, key findings from the results including conscious adaptation and planning are not new concepts that have been discussed in previous published literature. Therefore, the findings of the study lacked novelty. Unclear about how exploration of individual's experience with varying injuries severities can add to the literature. In addition, the discussion stated that conscious adaptation and planning were new findings but such concepts are well known in the literature related to traumatic injuries. The introduction and discussion need to highlight the novelty of this study.

Some additional comments for consideration:

Reflexivity can include the entire research group rather than just the two authors. Having perspectives of more than one profession may be helpful.

Understand that the topic guide was developed from a previous study but will benefit reader if the topic guide was also briefly summarised in the text of this paper. A description about this region of UK in the first paragraph of Methods will also be useful to understand the demographic characteristics of the participants.

Results will benefit from a coding tree.

VERSION 1 - AUTHOR RESPONSE

	Reviewer/editor comment	Response
	Reviewer 1	
1	The authors have explored patients' experience in the process of (major) injury recovery in a	Thank you for your feedback, we have

	qualitative manner. Although this design does not allow collecting evidence from a larger number of patients, it is indeed useful in terms of defining key themes, that can be further searched for in a quantitative manner. The paper has therefore merit and contributes to the understanding of patients' expectations and experience in the short term after injury. The following points could be addressed to provide more clarity:	addressed each of your points to further enhance the manuscript.
2	Why is the time interval of 6 months decisive according to the authors? One could argue that a time interval of 3 months - estimating 6 weeks of fracture healing and 6 subsequent weeks of gradual mobilisation could also be considered a turning point.	Thank you for your suggestion. We have chosen 6 months as typically this is where patients are more engaged with rehabilitation professionals as well as evidence suggesting that recovery does not necessarily follow healing time trajectories. We have made this clearer in the introduction on page 5.
3	Limited demographic information about the participants has been provided - certainly their age and biological sex distribution as well as type of injury are documented, however it would be helpful if the authors could provide more information enabling the reader to contextualise the social history and determinants of health (or disease) among the patients - eg their working status and whether they are in permanent or temporary employment, their residence in an urban/rural environment, their family status. These are representatives examples, but it may be understandable that data potentially leading to the identification of the patients cannot be presented.	Thank you for your suggestion. The editor had raised concerns with the demographic information we presented, and we have taken steps to change this (see point 3). Therefore, we do see the benefit of adding further detail re social history etc however we feel this would add further potential identifiable information and have chosen not to include further detail.
4	The issue/theme of financial (in)security has appeared. Discussing the insurance and social protection context in which the system supports patients would be useful for the reader - does for instance the inability to afford mortgage payments indicate that the salary of the patient was not (fully) subsidized or that the subsidy was consumed in the form of out-of-pocket payments, that could not be addressed in the frame of formal direct and indirect healthcare expenses?	Thank you for your suggestion. We have added a sentence on page 13 and further detail in the discussion on page 21 to highlight the complexity of the finance system within the UK and that the majority of healthcare is accessed through the

		national health service (NHS).
	Reviewer 2	
5	The manuscript covers a topic that is relevant and will be of interest to health professionals who worked with traumatic msk injuries. Considering the amount of current literature focusing on this topic, there appears to have quite a bit of published literature around emotion and physical factors influencing optimal rehabilitation at 6 months post injury.	Thank you for your feedback. We have made changes to the manuscript to make clear why this paper was needed with a focus to 6 months (introduction page 6, and discussion page 22). Further justification is included in point 10 below.<br><br>We hope our responses and additions to the manuscript has addressed your feedback and enhanced the manuscript.
6	Point 1<br>In addition, key findings from the results including conscious adaptation and planning are not new concepts that have been discussed in previous published literature. Therefore, the findings of the study lacked novelty.<br><br>Point 2<br>In addition, the discussion stated that conscious adaptation and planning were new findings but such concepts are well known in the literature related to traumatic injuries.	Thank you for your feedback around the concept of conscious adaptation and planning and the novelty of this paper.<br><br>We have amended the discussion to make clearer that the findings of this study go beyond just this one concept and show how this is related to how the patient as a whole responds to rehabilitation and calls for a more individualised approach to rehabilitation and recovery.<br><br>In response specifically around novelty. We accept and agree that there is other literature in this topic which focus on recovery. We have added additional references to include this

		within the manuscript to acknowledge this (page 20) and amended the wording of this section to make clearer.<br><br>Where this paper is different and novel is that the views were captured at 6 months specifically and not retrospectively or over a wide range of timepoints. This allows us to capture the challenges and views of patients and the lived experience at this vital timepoint in real-time representing clinical practice. We have tried to make this clearer throughout the manuscript e.g. introduction and strengths and limitations section.
7	Unclear about how exploration of individual's experience with varying injuries severities can add to the literature.	There is evidence to suggest that injury severity is not relevant to recovery and recovery trajectories therefore by widening the focus and looking beyond an injury type or severity reflects clinical practice and adding to the novelty of this paper.<br><br>We have added additional detail on page 5 to make this clearer.
8	The introduction and discussion need to highlight the novelty of this study.	We have made changes throughout the paper to highlight the novelty of this paper (see previous comments for specific examples).
9	Reflexivity can include the entire research group rather than just the two authors. Having	Thank you for this useful tip. Whilst we have not been able to gain the views

	perspectives of more than one profession may be helpful.	of other professions within this research we will endeavour to do this for future research. We have added a sentence in our limitations to reflect this (page 22).
10	Understand that the topic guide was developed from a previous study but will benefit reader if the topic guide was also briefly summarised in the text of this paper. A description about this region of UK in the first paragraph of Methods will also be useful to understand the demographic characteristics of the participants.	Thank you for this suggestion. We have added a copy of the topic guide as a supplementary document and given a brief overview as suggested on page 7. We have added a sentence about the description of region in the setting section on page 7.
11	Results will benefit from a coding tree.	We have added a coding tree as suggested.

VERSION 2 - REVIEW

Reviewer **1**

Name **Tsagkaris, Christos**

Affiliation **University of Zurich**

Date **19-Oct-2025**

COI

The authors have addressed my concerns

Reviewer **2**

Name **Tang, Clarice**

Affiliation **Victoria University**

Date **29-Oct-2025**

COI

Thank you for taking the time to address the responses.

I will agree that the changes made to the manuscript by the authors have strengthen the manuscript.

However, I have a couple of minor points to add.

1) The strengths and limitations section of the study on page 3 should include the limitation in which two of the researchers had very similar experiences and background and may have influenced the interpretation of the results.

2) minor error with the COREQ: In the response, authors stated that a coding tree was provided but the COREQ checklist suggest this was not done.

3)The presentation of participants quotes in the results section need to be address. Only two identifiers are required. For example: Male, 30-39 will be sufficient.

4) Manuscript requires a check for grammatical errors. Remained to be present throughout the manuscript. For example, Page 20, last paragraph: "Most literature, however, is focused to mild...". This sentence is grammatically incorrect.

VERSION 2 - AUTHOR RESPONSE

	Reviewer/editor comment	Response
	Reviewer 2	
1	The strengths and limitations section of the study on page 3 should include the limitation in which two of the researchers had very similar experiences and background and may have influenced the interpretation of the results.	This has been added.
2	Minor error with the COREQ: In the response, authors stated that a coding tree was provided but the COREQ checklist suggest this was not done.	Thank you for highlighting this omission, this has been updated.
3	The presentation of participants quotes in the results section need to be address. Only two identifiers are required. For example: Male, 30-39 will be sufficient.	This has been updated throughout the results.
4	Manuscript requires a check for grammatical errors. Remained to be present throughout the manuscript. For example, Page 20, last paragraph: "Most literature, however, is focused to mild...". This sentence is grammatically incorrect.	We have reviewed the manuscript for any grammatical errors and corrected these.
